# The Role of Trust in Text Messaging for Promoting Patient Portal Activation Among Low-Income Patients: Quality Improvement Project

**DOI:** 10.2196/80255

**Published:** 2026-05-04

**Authors:** Kevin Fiscella, Mechelle Sanders

**Affiliations:** 1 Department of Family Medicine, University of Rochester Medical Center, 1381 South Ave, Rochester, NY, 14620, United States, 1 585-324-4563

**Keywords:** text messaging, health information technology, digital divide, patient engagement, patient portal, mobile phone

## Abstract

**Background:**

The increasing reliance on patient portals for electronic health records has widened the digital health care access gap, particularly among low-income and Medicaid-insured populations. However, resources exist to assist low-income patients with portal enrollment; in obtaining a free smartphone; and, in New York, in obtaining low-cost internet. Automated bidirectional SMS text messaging offers a scalable and cost-effective strategy for identifying low-income patients’ digital health needs and eligibility for resources by using screening questions and providing tailored information on how to access available resources.

**Objective:**

This study aimed to increase portal access among low-income patients using automated bidirectional SMS text messaging and assess its feasibility and acceptability.

**Methods:**

This quality improvement initiative involved sending automated, bidirectional SMS text messages in English to 12,381 Medicaid-insured and/or low-income patients from a primary care practice. Messages assessed patients’ digital health needs and provided adaptive, personalized resources and assistance for enrolling in the patient portal and for accessing digital technology. We assessed response rates and follow-up portal enrollment rates. We surveyed participants regarding the acceptability, appropriateness, and usability of the SMS text messaging intervention, as well as their subsequent use of the patient portal. We performed descriptive statistics and a binomial probability test.

**Results:**

In total, 9.2% (1140/12,381) of patients responded to the SMS text messages, with 3.9% (481/12,381) opting out and 5.3% (659/12,381) actively engaging. Among respondents, 71.1% (469/659) completed the follow-up survey. Respondents were predominantly female (336/469, 71.6%), with ages ranging from 18 to 65 years or older. Most respondents rated the message’s clarity (420/469, 89.6%), its usefulness (400/469, 85.2%), and the demonstration of care by their health team (350/469, 74.6%) favorably. Concerns regarding privacy (61/469, 13%) and trustworthiness (71/469, 15%) were noted. Notably, 71% of initially unenrolled patients activated their patient portals after the intervention (*P*=.007), exceeding the hypothesized expectations.

**Conclusions:**

Automated bidirectional SMS text messaging had mixed effects on promoting patient portal use among low-income patients. Response rates to SMS text messages were low when delivered from an unknown phone number. Among responders, most reported that these messages were useful and that they would recommend them to others. Research is needed to determine optimal strategies for introducing the program and vendor phone numbers to patients to improve engagement.

## Introduction

The growing reliance on patient portals for electronic health records (EHRs) means that the existing digital divide creates a digital health care access divide [1]. Residence in a low-income zip code is often associated with lower enrollment in patient portals [2,3]. Frequent patient-level risk factors include age, education, limited digital health literacy, and affordability of digital technology [4,5]. Health care organizations can help low-income patients bridge this divide by training them to access and use patient portals and by connecting them to available digital resources for which they may be eligible, such as smartphones provided through the federal Lifeline program or affordable broadband internet in New York State; however, these interventions often require human navigators [6-8].

One promising approach for assisting low-income patients is the use of automated bidirectional SMS text messaging [9,10]. SMS text messaging interventions have been widely used with low-income patients [11,12]. This technology enables health organizations to deliver tailored information to likely eligible low-income patients [13], offering a scalable and cost-effective solution to promote engagement with digital health tools.

Automated bidirectional texting offers 2 theoretical advantages compared to automated unidirectional texting. First, it more closely resembles a human-to-human texting conversation, which may increase engagement. Second, it offers potential for personalization through adaptive responses that deliver relevant information to the individual based on branching logic from the previous response. This second advantage is particularly relevant in this context, where multiple eligibility categories exist for free smartphones or low-cost internet services [14,15]. Automated bidirectional texting can screen individuals and inform them that they are likely eligible and refer to social needs resources [16]. For example, being a recipient of Medicaid or the Supplemental Nutrition Assistance Program (SNAP) are standard requirements for eligibility for smartphones or low-cost internet services in New York State [14,15]. Finally, Gallup experiments found no demographic differences among SMS text messaging, SMS text messaging-to-web, and phone responders in terms of gender, race, or income level [17].

By identifying patients’ needs through SMS text–based questions and informing those who are eligible about available resources, including personal assistance, bidirectional texting offers a scalable, automated means to encourage patient portal use, provide help links, and share information on accessing free smartphones (eg, through community smartphone vendors) and low-cost internet services (eg, New York State programs and guidance to ask internet providers about US $15-US $20/month plans for eligible individuals).

We are not aware of previously published initiatives that promote access to digital health resources through automated bidirectional SMS text messaging [18]. However, there are a handful of studies that used SMS text messages to address other health-related social needs [18,19]. One protocol designed unidirectional SMS text messages to inform low-income, that is, federally qualified health patients, of resources to address those needs, for example, SNAP [18]. Another feasibility study used bidirectional SMS text messages to assess primary care patients’ needs for food, housing, and transportation using a link to 6 screening questions followed by tailored SMS text messages containing information regarding local resources based on screening questions and patient interest [19]. A third study screened patients in an emergency department for social benefits using bidirectional texting and referred those eligible to a benefits navigator [16].

We report on a quality improvement project involving the design and implementation of automated bidirectional SMS text messaging that directs patients with low incomes to personal assistance for patient portal enrollment and to resources for free smartphones and low-cost internet for qualifying individuals and families. Our specific objectives were to increase portal access among low-income patients by using automated bidirectional SMS text messaging and to assess the feasibility and acceptability of this strategy.

## Methods

As part of an internally funded quality improvement initiative through the Health Equity Program Services Office aimed at promoting patient portal use among low-income patients, we piloted automated bidirectional SMS text messages.

### Ethical Considerations

The Research Subjects Review Board of the University of Rochester approved the project as a quality improvement project. As required for privacy protection, identifying data, for example, names, phone numbers, and dates of birth, were removed from the data. Zip codes were converted to household median income using census data. Patients could opt out at any time by typing “STOP.” Participants who responded to the survey were entered into a lottery for a US $100 incentive, and 1 participant was selected. We followed the revised Standards for Quality Improvement Reporting Excellence (SQUIRE 2.0) (Checklist 1) [20].

### Practice Setting

The practice is part of an academic medical center in Western New York. It is staffed by primary care faculty, residents, nurse practitioners, and physician assistants and serves approximately 50,000 patients of all ages. The patient population is racially and ethnically diverse, with >35% insured through Medicaid. Patient portal “MyChart” activation among adults in the practice varied from the highest to the lowest quartile of zip code median household income: 85.4%, 80.9%, 77%, and 67.9%.

### Patient Sample

We obtained EHR data on patients in our primary care practice for the calendar year 2023. Eligibility criteria included being aged ≥18 years, having a practice encounter in the prior year, and being insured through Medicaid (including dual Medicaid and Medicare) or residing in a low-income zip code. Low-income residence was based on the 2022 American Community Survey 5-year estimates from the US Census Bureau and mapping these zip codes to categories based on annual median household income. These requirements for Medicaid or low household income, that is, <200% federal poverty, were based on the eligibility criteria for the Federal Communications Commission’s Lifeline program and/or the Affordable Connectivity Program (ACP) [21]. EHR data included patients’ age, sex, residential median household income by zip code, and phone number for texting.

### Bidirectional SMS Text Messages, Scripts, and Branching Logic

The authors (MS and KF) developed bidirectional SMS text messaging algorithms to assess patients’ digital health needs and offer tailored resources to meet those needs by using branching logic to create adaptive messages based on responses to prior texting questions. For example, we asked:

*Are you currently signed up for MyChart?*

*Yes→ We are glad to hear that*.

*No→ Are you interested in help in signing up for MyChart?*

*Yes→ Great. Do you have an email?*

*Yes→ Glad to hear it. If you don’t remember your username or password, you can get them by sending MyChart a message. Click here to make a request for your MyChart password and/or username*

We crafted SMS text messages for low-income patients where educational levels are low. SMS text messages were written in plain, concise English, with simple sentence structure (see Multimedia Appendix 1 for additional examples of SMS text messages). Terms such as patient portal vs MyChart were vetted with the committee advisory board. We used a series of questions to assess eligibility for these programs, but recognizing that some patients may not have wished to answer these sensitive questions even via a secure link, we also provided information to all patients who reported that they did not have a smartphone and were interested in obtaining one.

The initial SMS text message stated the following: “[Practice Name] is piloting text messages to share important information with our patients. You may choose to NOT receive these informational text messages at any time by typing STOP.” SMS text messages included questions about patient portal enrollment, awareness of the Lifeline smartphone program, and eligibility for the program [15], as well as tailored information on how to obtain assistance with portal enrollment, set up an email address, access the Lifeline program, and obtain low-cost internet services. Branching logic was used to tailor the information provided. We initially designed SMS text messages to promote awareness of the ACP but abandoned them when Congress failed to reauthorize funding for the ACP [22]. Instead, we substituted information on the New York State’s Affordable Broadband Act, which requires the large internet companies to offer internet services at affordable prices [23]. The SMS text messaging sequences were delivered in English twice over 3 weeks to each participant with an active phone number beginning February 25, 2025. The messages and their patterns are shown in Multimedia Appendix 1.

### Outcome Assessments

Outcomes included engagement (including at least 1 response to initial SMS text messages, an opt-out response, and the number of SMS text messages sent and acceptability based on responses to a survey administered through a link to Research Electronic Data Capture [REDCap; Vanderbilt University] [24]; see survey questions in Multimedia Appendix 1). We could not identify an existing scale to assess the constructs of interest, including trust in SMS text messages, comfort sharing sensitive information relevant to resource eligibility, and perceptions of the appropriateness of these SMS text messages from a primary care practice. Thus, we designed 6 survey items to evaluate patients’ perceptions of the acceptability, usefulness, and appropriateness of the SMS text messages using a 5-point Likert scale. Acceptability was based on how messages affected their perceptions of their primary care professional (PCP; “shows my healthcare team care...”) and whether they were understandable (“easy to understand”). We assessed usefulness directly (“the messages were useful”) and based on perceived value (“wish every medical office offered...”). Appropriateness was based on SMS text messages being perceived as “too personal” and trust (“did not trust these text messages”). The SMS text messages were written at a seventh-grade reading level based on the Flesch Kincaid Calculator [25]. The survey items were brief to optimize the response rate. Items used plain, simple questions to enhance understandability and were also written at a seventh-grade reading level based on the Flesch Kincaid Calculator [25]. The survey was delivered via a link embedded in an SMS text message 3 weeks after the intervention. Responses were dichotomized to simplify analysis.

### Data Analysis

We analyzed the data using REDCap [26] and Stata (version 18; StataCorp), and we considered *P* values <.05 statistically significant. We compared completed categorical responses using chi-square or Fisher exact tests (for responses <5). We reported missing data and did not use imputation. We used a binomial test to assess whether the observed proportion of participants who activated their portal after the texting intervention differed significantly from the hypothesized proportion of 0.5. This test is appropriate when the outcome is binary, and the hypothesis is a proportion or probability of “success.” The 0.5 estimate was based on studies of in-person and phone assistance in portal enrollment with low-income patients that achieved roughly 60% enrollment [27,28].

## Results

### Characteristics of the Sample

The characteristics of the sample are shown in Table 1. The population was slightly skewed toward females (7181/12,381, 58%) and showed a broad age range (<30 to >65 years). Median annual household income by zip code reflected patients who were insured by Medicaid or who resided in low-income areas, with about half of the full sample living in zip codes with median household incomes <US $30,000 and with 1 in 4 not having an active patient portal account. As expected, there was a statistically significant graded relationship between residence in a low-income zip code and portal enrollment (*P* value for trend <.001), with rates of 69.5%, 75.7%, 83.5%, and 84.7% for zip codes with median annual household incomes <US $30,000, US $30,000 to US $39,999, US $40,000 to US $49,999, and US $50,000 to US $59,999, respectively.

In total, 9.2% (1140/12,381) of patients responded to the SMS text messages, including 3.9% (481/12,381) who texted “STOP,” which terminated any further SMS text messages. This left 5.3% (659/12,381) of patients who responded to any message. Compared with the full sample in Table 1, survey respondents differed significantly in their characteristics (*P*<.001), being more likely to be female and older, less likely to reside in the lowest-income zip codes, and more likely to be enrolled in the patient portal. The mean and median number of SMS text message responses to these automated messages was 4.5 (SD 8.79) and 4.0, respectively, with an interquartile range of 2.0-5.0.

**Table 1.** Characteristics of the full sample of patients receiving SMS text messages (N=12,381).

	Texting invitees, n (%)
Sex	
Female	7181 (58)
Male	5196 (42)
Missing	4 (0)
Age (years)	
<30	2352 (19)
30-45	5204 (42)
46-64	3215 (26)
≥65	1610 (13)
Missing	0 (0)
Median annual household income by zip code (US $)	
<30,000	6204 (50.1)
30,000-39,999	658 (5.3)
40,000-49,999	3234 (26.1)
50,000‐59,999	1901 (15.3)
>60,000	0 (0)
≥70,000	—^a^
Missing	384 (3.1)
Portal active	
Yes	9384 (75.8)
Inactive or unknown or missing	2997 (24.2)

a Not applicable.

### Survey Responses

In total, 71.1% (469/659) of those who responded and did not opt out completed the postintervention survey. Compared with the full sample of patients who received any SMS text message, patients completing the survey (Table 2) had a greater proportion of women, were more often aged >65 years, and were less likely to reside in the lowest-income zip codes.

Survey respondents reported favorably on the SMS text messages (Table 2). For example, roughly three-quarters (350/469, 74.6%) agreed or strongly agreed that the SMS text messages showed the health care team cared about them and wished all medical offices offered this service (360/469, 76.8%). The vast majority (420/469, 89.6%) reported that the SMS text messages were easy to understand and found the information useful (420/469, 89.6%). Respondents were slightly less favorable about questions being personal or trusting the messages. While most respondents disagreed or strongly disagreed that the messages were too personal to answer (403/469, 85.9%) or that they did not trust the messages (392/469, 83.5%), a small minority of respondents agreed or strongly agreed (61/469, 13%) that they were too personal or that they did not trust them (71/469, 15.1%).

The results were stratified by whether the individual had an active patient portal account or not (Table 2). Those without portal access were more often male aged (*P*<.001) and were also significantly less likely to report not qualifying for a free phone (*P*<.001), but there were no statistically significant differences in survey responses, except for lower trust among those without baseline portal activation (*P*=.03).

**Table 2.** Characteristics of survey respondents by patient portal activation status.^a^

Characteristic			Full sample (n=469), n (%)	Baseline nonactive patient portal (n=45), n (%)	Baseline active patient portal (n=424), n (%)	*P* value
Sex						<.001
Female			319 (68)	14 (31.1)	305 (71.9)	
Male			150 (32)	131 (68.9)	119 (28.1)	
Missing			0 (0)	0 (0)	0 (0)	
Age (y)						.07
<30			68 (14.5)	12 (26.7)	56 (13.2)	
30‐45			150 (32)	6 (13.3)	1144 (34)	
46‐64			170 (36.2)	16 (35.6)	154 (36.3)	
>65			81 (17.3)	11 (24.4)	70 (16.5)	
Missing			0 (0)	0 (0)	0 (0)	
Median annual household income by zip code (US $)						.38
<30,000			68 (14.4)	5 (11.1)	63 (14.8)	
30,000‐39,999			108 (23)	15 (33.3)	93 (21.9)	
40,000‐49,999			162 (34.5)	16 (35.6)	146 (34.4)	
≥50,000			75 (16.2)	5 (11.1)	70 (16.5)	
≥60,000			—^b^	—	—	
Missing			56 (11.7)	4(8.9)	52 (12.7)	
Use of free Lifeline smartphone						<.001
Never heard of it			158 (33.7)	16 (38)	142 (33.4)	
Aware of the program but not interested			66 (14.1)	7 (17)	59 (13.9)	
Aware and interested but have not applied			77 (16.4)	8 (19)	68 (16)	
Applied but did receive it			29 (6.2)	2 (5)	27 (6.4)	
Currently using one			34 (7.2)	4 (10)	30 (7.1)	
I do not qualify			101 (21.5)	4 (9)	97 (22.9)	
Missing			2 (0)	3 (7)	2 (0.5)	
Sending SMS text messages shows that my health care team cares about me						.19
Strongly agree or agree			350 (74.6)	37 (82)	313 (73.8)	
Strongly disagree or disagree or neutral or unsure			111 (23)	8 (18)	103 (24.2)	
Missing			8 (1.7)	0 (0)	8 (2.9)	
These SMS text messages were easy to understand						.77
Strongly agree or agree			420 (89.6)	40 (89)	380 (89.6)	
Strongly disagree or disagree or neutral or unsure			43 (9.1)	5 (11)	38 (9)	
Missing			6 (1.2)	0 (0)	6 (1.4)	
The information in the SMS text messages was useful						.69
Strongly agree or agree			400 (85.2)	39 (87)	361 (85.1)	
Strongly disagree or disagree or neutral or unsure			51 (10.8)	6 (13)	46 (10.8)	
Missing			7 (1.5)	0 (0)	7 (1.6)	
Some of the questions were too personal to answer						.06
Strongly agree or agree			61 (13)	10 (22)	51 (12)	
Strongly disagree or disagree or neutral or unsure			403 (85.9)	35 (78)	368 (86.8)	
Missing			5 (1.1)	0 (0)	5 (1.2)	
I did not trust the SMS text messages						.03
Strongly agree or agree			71 (15.1)	12 (27)	59 (13.9)	
Strongly disagree or disagree or neutral or unsure			392 (83.5)	33 (73)	359 (84.7)	
Missing			7 (1.5)	1 (2)	6 (1.4)	
I wish every medical office offered this service to patients						.24
Strongly agree or agree			360 (76.8)	38 (84)	322 (76)	
Strongly disagree or disagree neutral or unsure			105 (22.4)	7 (16)	98 (23)	
Missing			4 (0.9)	0 (0)	4 (1)	
Follow-up use of the patient portal						<.001
Not enrolled			16 (3.4)	12 (27)	4 (1)	
Enrolled but have not used it			12 (2.6)	8 (18)^c^	4 (1)	
Enrolled but use it once			10 (2.1)	0 (0)	10 (2)	
Enrolled and used it more than once			430 (91.7)	24 (53)^c^	406 (96)	
Missing			5 (1.1)	3 (7)	2 (0.5)	

a *P* values were derived from chi-square tests. Survey items were dichotomized as strongly agree or agree vs all other responses. Summed percentages may not always equal 100% due to rounding. The increase in portal activation (from 0 at baseline) was statistically significant based on a hypothesized proportion of 50% (*P*<.001, binomial test).

b Not applicable.

c The person activated the account but did register a login.

### Portal Activation

Among the 45 participants who were not enrolled in the portal at baseline (December 2023), 33 (71%) had activated their portal by the 3-week follow-up (March 19, 2025). This differed significantly from our conservative estimate of 50% (*P*=.007 by the binomial probability test). Among those who reported portal activation, 3 in 4 patients reported using the portal.

## Discussion

This quality improvement project illustrates both the challenges and opportunities of using automated bidirectional texting to promote portal use among low-income patients.

### Patient Portal Activation and Use

Our primary objective was to promote patient portal use using SMS text messaging. Among patients without portal activation who responded, 71% (3245) did so, and three-fourths (24/32) reported using it. These findings are consistent with those previously reported using more intensive interventions. A 2019 systematic review of interventions to increase portal use in vulnerable populations found that 12 of 18 studies reported a significant increase in portal use, with enrollment ranging from no improvement to 48% of patients enrolling [29]. Subsequent studies have reported higher portal enrollment rates. In an initiative that deployed patient navigators at the point of care at federally qualified health centers, 64% of patients enrolled among those who accepted navigator assistance [27]. The Digital Access Coordinator program involved 12 multilingual navigators who supported patients in enrolling and using the patient portal across a large primary care network in Boston [28]. Navigators reached out by phone to 16,045 patients; 84% were contacted, and 61% of those contacted were successfully enrolled in the patient portal [28]. However, in contrast to these findings, our findings are limited by low initial engagement with SMS text messages.

### SMS Test Message Engagement

Engagement with the messages was modest: only 9.3% (1140/12,381) of patients responded to any message, including 3.9% (481/12,381) who opted out by testing “STOP.” Furthermore, patients who were not enrolled in the portal were significantly less likely to respond to SMS text messages than those who were enrolled (*P*<.001). The 9.3% (1140/12,381) response rate is roughly similar to the 8.5% reported by Kormanis et al [19], but contrasts sharply with a response rate of 62% reported by Bruce et al [30] among 78,883 patients discharged from a large Texas hospital. Engagement thus represents a core challenge. We had planned to use the practice phone number, but we were required to use the vendor phone number. In retrospect, we recognize our error in not engaging primary care providers sufficiently or using other means to alert patients to these SMS text messages through practice handouts. Among patients who elected to engage, most responded to the sequence of SMS text messages, as indicated by the mean and median numbers of texts sent. Nonetheless, these findings are a warning sign for others who do not adequately prepare patients for SMS text messages from unknown sources.

### Acceptability

The positive reception of the messages among users, particularly perceptions of caring, ease of understanding, and perceived usefulness, underscores the acceptability of automated texting as a modality for engaging underserved populations in digital health. Our results suggest that thoughtfully designed SMS text messaging campaigns, when paired with tailored resources and referrals for assistance, may promote enrollment in the portal and access to enabling technology such as free smartphones and low-cost broadband internet.

### Trust

While 13% (61/469) to 15% (71/469) of respondents indicated that the messages felt too personal or were not trustworthy, it is plausible that a greater proportion of nonresponders did not trust the source of these messages than those who responded. Prior research suggests that lack of trust in the source of the SMS text messages may have contributed to the very low overall response to these bidirectional SMS text messages. Experimental findings show that requests from a familiar sender yield improved survey response rates (54.3%) compared to those from an unfamiliar sender (36.9%; odds ratio 2.03, 95% CI 1.23-3.33) [31]. An experimental study of real and fake messages showed that more than half of the time users believed real SMS text messages were fake, and participants who were younger and had lower income were less likely to interact [32]. Potentially, differences in trust in the SMS message source may account for the striking differences in response rates between our study and those reported by Bruce et al [30], which observed a very high response rate to automated bidirectional SMS text messaging. Notably, the key distinction was that the patients in the study by Bruce et al [30] had consented to receive SMS text messages and were alerted by the hospital discharge team to forthcoming SMS text messages. These steps by Bruce et al’s [30] study team plausibly built trust among patients motivated to obtain key information postdischarge. Moreover, the high and growing prevalence of phishing scams via SMS text messages likely further undermines trust in SMS text messages from unknown phone numbers [33,34].

### Policy Adaptation

Adoption of trusted phone numbers for automated texting poses a major challenge. Most US or Canada phone carriers now treat most automated or clinical texting as application-to-person traffic, meaning that these messages are expected to originate from phone numbers that are delivered using the vendor’s messaging infrastructure and correctly registered or verified [35]. Furthermore, it is simplest and more reliable when the “from” number is a number the vendor controls within their messaging stack. This poses a barrier to indicating that the SMS text message originates from the known organization’s phone number that is familiar to patients.

In the absence of policy, regulatory, systems, and verification reforms, health care organizations may consider several options to improve trust in automated SMS text messages sent from unfamiliar numbers. First, the organizations can promote texting programs and numbers through flyers and postings. Second, they can deploy intermediaries, for example, receptionists, nurses, care managers, or PCPs, who introduce the program and encourage patients to add the vendor’s number to their phone contacts. However, this labor-intensive option reduces scalability. Third, the health care organization can use intermediary messages delivered automatically through the patient portals. This option is not helpful for patients who are not yet using their portal. Finally, simple verification systems have been developed to verify SMS text messages [36]. Thus, multiple options may be needed to promote awareness and trust in SMS text messages among diverse patients.

### Limitations

Our findings should be interpreted in the context of several limitations. This was a nonrandomized, single-arm quality improvement project without a control group, limiting causal inference. Furthermore, SMS text messages were delivered only in English, excluding patients with limited English proficiency. We assessed a limited number of demographic characteristics. Our survey was not psychometrically validated. There was evidence of response bias. Patients who responded to the survey differed from the full sample in age, sex, median income, and portal enrollment at baseline, potentially overestimating acceptability, usefulness, and appropriateness, thereby reducing generalizability. Notably, respondents were less likely to reside in the lowest-income zip codes and more likely to be enrolled in the portal than nonrespondents, suggesting limitations in reaching patients in the lowest-income zip codes who lacked portal access. There was a 14-month delay between sample extraction and survey completion, during which some patients without portal enrollment may have enrolled before texting. However, a review of changes in overall portal enrollment showed a 2% to 3% increase, compared to our assumption of a 50% increase.

### Conclusions

Bidirectional automated SMS text messaging offers a possibly feasible, acceptable, and scalable strategy for promoting patient portal activation and access to relevant technology among Medicaid-insured and low-income individuals. However, further research is needed to determine optimal strategies for introducing the texting program to low-income patients, reaching patients in the lowest-income areas, and promoting trust in forthcoming SMS text messages.

## Supplementary material

10.2196/80255 Multimedia Appendix 1 Text messages branching logic.

10.2196/80255 Checklist 1 SQUIRE Checklist.

## Abbreviations

ACP: Affordable Connectivity Program
EHR: electronic health record
PCP: primary care professional
REDCap: Research Electronic Data Capture
SNAP: Supplemental Nutrition Assistance Program
