# Supplementary material for: The Role of Trust in Text Messaging for Promoting Patient Portal Activation Among Low-Income Patients: Quality Improvement Project
Source: JMIR Form Res. 2026 May 4;10:e80255. doi: 10.2196/80255 (PMC13138706; doi:10.2196/80255)
Supplement: Multimedia Appendix 1 [file formative-v10-e80255-s001.pdf]

Supplementary File

Text Messages and REDCAP Integration for MYCHART and LIFELINE PROMOTION

Message A1. (Introduction):

[practice NAME] is piloting text messages to share important information with our patients. You may choose to NOT receive these informational text messages at any time by typing STOP.

Message A2.
Do you have a phone that allows you to access the internet?

Yes→Go to message A3

No→Go to Message B1

Message A3:
We encourage all patients to use MyChart. This allows you to message your team, and to see your test results. We want to make sure that all our patients have access to MyChart and equipment for video visits. Please respond to the following confidential questions so we provide you with information that matches your needs.

Are you currently signed up for MyChart?

Yes→ We are glad to hear that. Do you have any questions about to best use it?

Yes→ If you would like someone to call you, please type your number and good times for staff to call you

No → Keep using it!

No→Are you interested in help in signing up for MyChart?
Yes→ Great. Do you have an email?
Yes→Glad to hear it. If you don't remember your username or password, you can get them by sending MyChart a message. Click here to make a request for your MyChart password and/or username

No→ You can sign up for gmail here. Once you an email, you can request a usernamer and password by sending MyChart a message. Click here to make a request for your MyChart password and/or username

No→go to B1

Message B1. Sends 2 weeks after A (if ppt branches here)

Do you have a computer or tablet that can access the internet?

Yes→B2

No→B3

Message B2.
Are you currently signed up for MyChart?
Yes→ We are glad to hear that. Do you have any questions about to best use it?

Yes→ If you would like someone to call you, please type your number and good times for staff to call you

`No → Keep using it!

No→Are you interested in help in signing up for MyChart?
    Yes→Do you have an email?
Yes→Glad to hear it.
You can call 1-888-661-6162, Opt. 1 or type https://mychart.urmc.rochester.edu into your web browser to get your username or password.

No→ You can sign up for gmail here. Once you have an email, You can call 1-888-xxx-xxxx, Opt. 1 or type https://mychart.xx.xxx.edu into your web browser to get your username or password.

No→ If you change your mind click here to return for how to do it

Messages B3:
(First message)
Librarians at public libraries can help access the internet in the library or using a loaner hub. Click here to learn more about how to loaner hubs from the Rochester library.

(2nd message 24 hours later) (Not doing – will send at same time)
You might be eligible for a free smartphone and minutes. Are you interested in learning if you're eligible?
Yes→B4 (goes to B5)

No → Please text: R if you change your mind. (R goes to B5)

Message B4:
Many patients don't know they are eligible for a free smartphone and minutes. Would you like to find out if you qualify?

Yes→B5

No→ If you change your mind, please type R   (goes to B5)

Messsage B5 (To all unless they sent STOP one week after last message)

Please click on the following link to take a short confidential survey to determine your eligibility:
<link>

You can qualify for free smartphones and free minutes in two ways. The first is participation in government programs and the second is based on household income. We will help determine if you qualify. Please answer the following questions about government programs.
Do you have Medicaid usually provided through Blue Choice, Fidelis, MVP or United?
Yes
No
Do you receive SNAP often called food stamps?
Yes
No
Do you receive SSI benefits?
Does anyone in your household participate in the National School Lunch Program?
Yes
No
Do you receive SSI benefits?
Yes
No
Do you receive veterans' pension or survivors' benefits?
Yes
No
Do you receive temporary assistance for needy families formerly called Welfare?
Yes
No
Do you receive Tribal program benefits for Native Americans?
Yes
No

If any Yes→You likely qualify for free smartphone/minutes→B6

If Yes to SSI or you have a child who receives free school lunch, you may qualify for Spectrum's $29.99 month internet assist program.
Are you interested in calling Spectrum?
    Yes→ call xxx xxx-xxxx
    No (skip to next question)

If all No→You might qualify based on your household income. Please answer the following questions.
How many people are in your household?
1
2
3
4
5
6
7
8

If 1→Is your annual household income below $19,683 or $1640 per month?
Yes→You likely qualify for the lifeline (Obama smartphones)
No→It does not appear you qualify

If 2→Is your annual household income below $26,622 or $2,219 per month?
Yes→You likely qualify for the lifeline (Obama smartphones)
No→It does not appear you qualify

If 3→Is your annual household income below $33,561 or $2,797 per month?
Yes→You likely qualify for the lifeline (Obama smartphones)
No→It does not appear you qualify

If 4→Is your annual household income below $40,000 or $3,375 per month?
Yes→You likely qualify for the lifeline (Obama smartphones)
No→It does not appear you qualify

If 5→Is your annual household income below $47,439 or $3,953 per month?
Yes→You likely qualify for the lifeline (Obama smartphones)
No→It does not appear you qualify

If 6→Is your annual household income below $54,378 or $4,532 per month?
Yes→You likely qualify for the lifeline (Obama smartphones)

No→It does not appear you qualify
If 7→Is your annual household income below $61,317 or $5,110 per month?
Yes→You likely qualify for the lifeline (Obama smartphones)
No→It does not appear you qualify

If 8→Is your annual household income below $68,256 or $5,688 per month?
Yes→You likely qualify for the lifeline (Obama smartphones) Yes→ B6.

No→It does not appear you qualify

If Yes→You likely qualify for a free smartphone and limited monthly minutes. Are you interested in learning more for yourself or family members?
Yes→ B6.
No→If you change your mind, text R to return for information on obtaining a free smartphone

Message B6.
Below are companies that offer this program with their phone numbers. You can click on links to their website to read about what they offer or call using the numbers listed below to sign up.

Safelink Wireless 800-723-3546

Access Wireless 866-594-3644

Assurance Wireless 888-898-4888

Infiniti Mobile 888-801-0012

Spectrum 833 224-6603

Message B7.
Do you plan to call and sign-up?

Yes → Great!. We hope this program saves you money.

No→ You can can click here to learn more about the program or call this number to get more information 800-234-9473

Message B8. (72 hours after last MyChart message).
Your Highland Family Medicine team is checking back about MyChart. Please respond to the following confidential questions so we can provide you with information that matches your needs.

Were you able to sign in to MyChart?

Yes→Congratulations! We hope you find it useful

No→ would you like to request your MyChart password or username?
- Yes. Click here to make a request for your MyChart password and/or username
- No→Would you like a staff member to call you?
  - Yes→Type in your name and best day/time to be called.
  - No→Type R if you change your mind (go back to the YES branch above)

Message C1 (One Three weeks after last text message)
Highland Family Medicine is conducting a patient survey about receiving text messages from us about using MyChart and free smartphones. We are offering participants a chance to win $200 through a lottery among participants.

To participate, please click on this [survey link] to ensure your responses are confidential→C2

(Need to get the survey link)

C2. POST-TEXTING SURVEY
Please indicate which best describes your use of MyChart

_I am not enrolled in MyChart
_I am enrolled in MyChart but have not used it
_I am enrolled in MyChart and have used it one time
_I am enrolled in MyChart and have used it more than once

Please indicate which best describes your use of the free smartphone through the lifeline

_I have never heard of this free program.
_I am aware of this program for free smartphones, but am not interested.
_I am aware of this program for free smartphones, and I am interested but have not applied.
_I applied but I did not receive a free smartphone.
_I am currently receiving a free smartphone.
_ I do not qualify for this free smartphone program

Please respond to the following questions with how much you agree 5= high agreement 1= disagree

It is helpful for Highland Family Medicine to send information to patients using text messages.
5 Strongly agree
4 Agree
3 Neutral/Unsure
2 Disagree
1 Strongly disagree

Sending these text messages shows that my healthcare team cares about me.

5 Strongly agree
4 Agree
3 Neutral/Unsure
2 Disagree
1 Strongly disagree

The messages were easy to understand.

5 Strongly agree
4 Agree
3 Neutral/Unsure
2 Disagree
1 Strongly disagree

The information in the messages was useful.
5 Strongly agree
4 Agree
3 Neutral/Unsure
2 Disagree
1 Strongly disagree

Some of the questions were too personal to answer.
5 Strongly agree
4 Agree
3 Neutral/Unsure
2 Disagree
1 Strongly disagree

I did not trust these text messages.
5 Strongly agree
4 Agree
3 Neutral/Unsure
2 Disagree
1 Strongly disagree

I wish every medical office offered this service to patients.

5 Strongly agree
4 Agree
3 Neutral/Unsure
2 Disagree
1 Strongly disagree
